# A study on atypical Kashin–Beck disease: an endemic ankle arthritis

**DOI:** 10.1186/s13018-023-03633-8

**Published:** 2023-05-02

**Authors:** Fang Qi, Si-Lu Cui, Bing Zhang, Hao-Nan Li, Jun Yu

**Affiliations:** 1 Institute for Kashin-Beck Disease Control and Prevention, Chinese Center for Disease Control and Prevention, Harbin Medical University, Harbin, 150081 Heilongjiang China; 2 National Health Commission and Education Bureau of Heilongjiang Province, Key Laboratory of Etiology and Epidemiology, Harbin Medical University(23618504), Heilongjiang Provincial Key Laboratory of Trace Elements and Human Health, Harbin Medical University, Harbin, 150081 China

**Keywords:** Kashin–Beck disease, Ankle changes, Atypical Kashin–Beck disease

## Abstract

**Background:**

To study the epidemiological characteristics of atypical Kashin–Beck disease cases without characteristic hand lesions such as interphalangeal joint enlargement and brachydactyly and the characteristics of ankle joint lesions.

**Methods:**

We investigated Kashin–Beck in the endemic villages in Heilongjiang Province. The patients were judged according to the “Diagnosis of Kashin–Beck Disease” (WS/T 207–2010). The severity of foot lesions was judged based on the changes of X-ray images. Residents of non-Kashin–Beck disease area were selected as normal controls in Jilin Province.

**Results:**

A total of 119 residents over 40 years old were surveyed in a natural village in the non-endemic area. A total of 1190 residents over 40 years old were surveyed in 38 endemic areas of Kashin–Beck disease. A total of 710 patients with Kashin–Beck disease were detected, including 245 patients with grade I, 175 patients with grade II, 25 patients with grade III, and 265 atypical patients. Among all investigated patients, 92.0% (653/710) had ankle joint changes, and it was 80.0% (196/245) in grade I patients and 95.4% (167/175) in grade II. Varying degrees of ankle joint changes were found in both grade III and atypical patients. The grade of Kashin–Beck disease was correlated with the degree of ankle joint change (*P* < 0.001), and the correlation coefficient *r*_*s*_ = 0.376. Atypical Kashin–Beck disease patients in mild and severe endemic area of Kashin–Beck disease were younger than those with typical Kashin–Beck disease.

**Conclusions:**

We found a correlation between the degree of ankle joint change and the grade of Kashin–Beck disease. The higher the grade of Kashin–Beck disease, the more serious the change of the ankle joint.

## Background

Kashin–Beck disease (KBD) is an endemic, deforming osteoarthropathy. New cases of KBD occur mainly in childhood and adolescents. It is found primarily in agricultural regions of eastern Siberia, northern Korea, and the central regions of China [1]. In China, it involves Heilongjiang, Jilin, Liaoning, Hebei, Shandong, Shanxi, Henan, Shaanxi, Inner Mongolia, Gansu, Qinghai, Sichuan, Tibet provinces, and autonomous regions. The narrow strip from Sichuan and Tibet to the northeast, extending to eastern Siberia in Russia and a few areas in northern North Korea [2, 3]. The etiology and pathogenesis of KBD have not been fully elucidated. The combination of low protein intake, polluted grain, and selenium deficiency may contribute to be onset of KBD together [4, 5]. Multiple symmetrical degeneration and necrosis of epiphyseal cartilage, articular cartilage during growth and development are the primary lesions of Kashin–Beck disease, and it will result in secondary degenerative osteoarthropathy [6]. The pathogenesis includes articular cartilage damage, chondrocyte apoptosis, and proliferative inflammation [7, 8]. Clinically, the manifestations are as follows, joint pain, thickening, deformation, limited mobility, and muscle atrophy in the limbs. Brachydactyly (toes), short limbs, or even dwarfism may occur in severe cases [9]. “Short fingers, enlarge joints, dwarfism, and curved arms,” “big heels, bulging the soles of the feet, just like with a basket on his arm, and a wiggle on his hips” are to describe the appearance of typical KBD cases.

According to the survey results of China's endemic disease in 2020, more than 170,000 patients with Kashin–Beck disease exist in China, and 20 million people in wards are threatened by the risk factors of KBD, most of the patients are over 50 years old. KBD seriously affect physical, psychological health and social aspects of patients, and causes serious economic burden to their families and society [10]. In China, although the prevalence of KBD has reached the level of elimination or control in the majority of affected areas, the loss of health life due to KBD should not be ignored [11]. The pain and limited range of joint were the independent risk factors of daily living and working function [12].

The diagnosis of Kashin–Beck disease is usually based on the life history in the KBD area, clinical symptoms, and X-ray changes in hand [13]. The current clinical classification of KBD diagnostic criteria is mainly divided into grade I, grade II, and grade III based on changes in the hand. The bone damage of the whole body in patients with KBD is mostly symmetrical, mainly in the limbs and joints. The milder only affects the hands, wrists, or feet and ankles. The moderate ones affect the elbows and knees, and the severe ones affect the shoulders, hips, and spine joints. However, only ankle joint damage in some patients in endemic areas, there is no typical change in the hand. These patients were called atypical KBD patients, and few literature reported the characteristics of the disease. In order to study the characteristics of atypical KBD, we carried out this research. Meanwhile, the damage characteristics in ankle joint of KBD, and whether it is consistent with the degree of KBD are also the focus of our research.

### Objects and methods

#### Investigation location

The cross-sectional study was used in this study. A total of 39 natural villages were selected as investigation sites in the historically severe, moderate, mild KBD areas, and non-endemic areas. In severe endemic areas of KBD, two natural villages were selected in Yanshou County, 20 natural villages in Fuyu County, and 10 natural villages in Shangzhi City of Heilongjiang Province. In moderate endemic areas of KBD, 1 natural village were selected in Tieli City, 1 natural village were selected in Bei'an City, and 2 natural villages were selected in Ning'an City in Heilongjiang Province. Two natural villages were selected in Acheng District of Harbin, a historically mild disease area of KBD. A natural village in Qian'an County, Jilin Province was selected as control.

#### Survey object and survey content

Questionnaire survey, clinical examination, and posterior-anterior of both hand and ankle-lateral X radiographs were conducted among villagers over 40 years old in the warded villages. The contents of the questionnaire included gender, age, age of onset, and previous medical history.

### Diagnosis and diagnosis criteria

#### Determination and division of Kashin–Beck disease areas

Refer to the Standards for “Determination and Division of Kashin–Beck Disease Areas” (GB16395-2011) [14]. Those with the following two conditions were judged as KBD areas: ①. More than 5% of the local residents were KBD clinical grade I and above, reached epidemic levels. ② A case of 7–12 years old children with multiple and symmetric changed in distal end of phalanx according hand X-ray. According to the severity of the endemic areas, it was divided into three conditions. ① Mild endemic area of KBD: The prevalence of clinical grade Ӏ and above of local residents or the X-ray detection rate of children aged 7–12 years is ≤ 10%. ② Moderate endemic area of KBD: The prevalence of clinical grade I and above in local residents or the X-ray detection rate of children aged 7–12 years is > 10% and ≤ 20%. ③ Severe endemic area of KBD: The prevalence of clinical grade I and above in local residents or the X-ray detection rate of children aged 7–12 years is > 20%.

### Diagnostic criteria for KBD

Diagnostic criteria for KBD: refer to the “Diagnosis of Kashin–Beck Disease” standard (WS/T 207–2010) [15, 16]. KBD cases can be diagnosed according to the contact history of the ward, symptoms and signs, as well as the multiple symmetrical depressions, sclerosis, destruction and deformation of the metaphyseal pre-calcification zone changes of metacarpophalangeal bones, osseous articular surfaces of wrist joints, as well as other changes in hand X-ray films, and excluded other related diseases. Multiple symmetrical changes in the distal phalanx are characteristic of the disease. Grade I: Multiple and symmetrical thicken finger joints, thickening of other limbs, limited flexion and extension activities, pain, and mild muscle atrophy. Grade II: On the basis of grade I, symptoms and signs are aggravated, and brachydactyly (toe) deformity occurs. Grade III: On the basis of grade II, symptoms and signs are aggravated, and dwarfism appear.

### Degree of ankle joint changes criteria

In this study, ankle joint space narrowing, articular surface irregularity, osteophyte formation, and subchondral bone sclerosis were determined as changes in the ankle joint. A classification standard for ankle joint changes was established according to the ankle joint changes of the patients in this study (Table 1).

**Table 1:** Grading scale of ankle joint changes

Degree classification	Grading standards
Normal	The calcaneus is normal in length. No hyperplasia of the talar head and body. The articular surface of the talus is smooth
Degree I	The calcaneus is slightly shorter. The talar head and talar body proliferate. The articular surface of the talus is smooth
Degree II	The calcaneus is slightly shorter. The talar head and talar body proliferate. The articular surface of the talus is rough and uneven
Degree III	The calcaneus is short. The talar head and talar body proliferate. The articular surface of the talus is collapsed

### Testing equipment

High-frequency portable digital medical diagnostic X-ray image DR system (Langan Image Technology Co., Ltd, Beijing, China).

### Detection method

DR detection sites are metacarpophalangeal joints and ankle joints. Shooting pose: ①Back and front position photography with both hands: the patient sits next to the examination bed, with the forearm lying flat on the examination table, and palms facing down on the image receiver. Position the center of the image receiver at the metacarpophalangeal joint. And the long axis of the receiver is perpendicular to the long axis of the hand and forearm. The patient relaxes the hand and spreads the fingers slightly to avoid movement of the palm. ②Lateral ankle photography: The patient was in a lateral position. The long axis of the lower limb is parallel to the long axis of the image receiver. The lateral surface of the foot is in contact with the image receiver. And the back of the foot is stretched to keep the angle between the sole of the foot and the lower limbs at 90° to prevent the rotation of the ankle joint.

### Statistical method

SPSS software 25.0 was used for statistical analyses. The Chi-square test (*χ*^2^) was used to compare the rates of ankle changes. The Spearman rank correlation was used to analyze the correlation between the grade of KBD and the degree of ankle joint changes. Ages of typical patients and atypical patients in each area were expressed as median and quartile. Age distributions were compared using the Mann–Whitney test. *P* < 0.05 was considered statistically significant.

## Results

### General information and detection rate of KBD patients

**Fig. 1:**
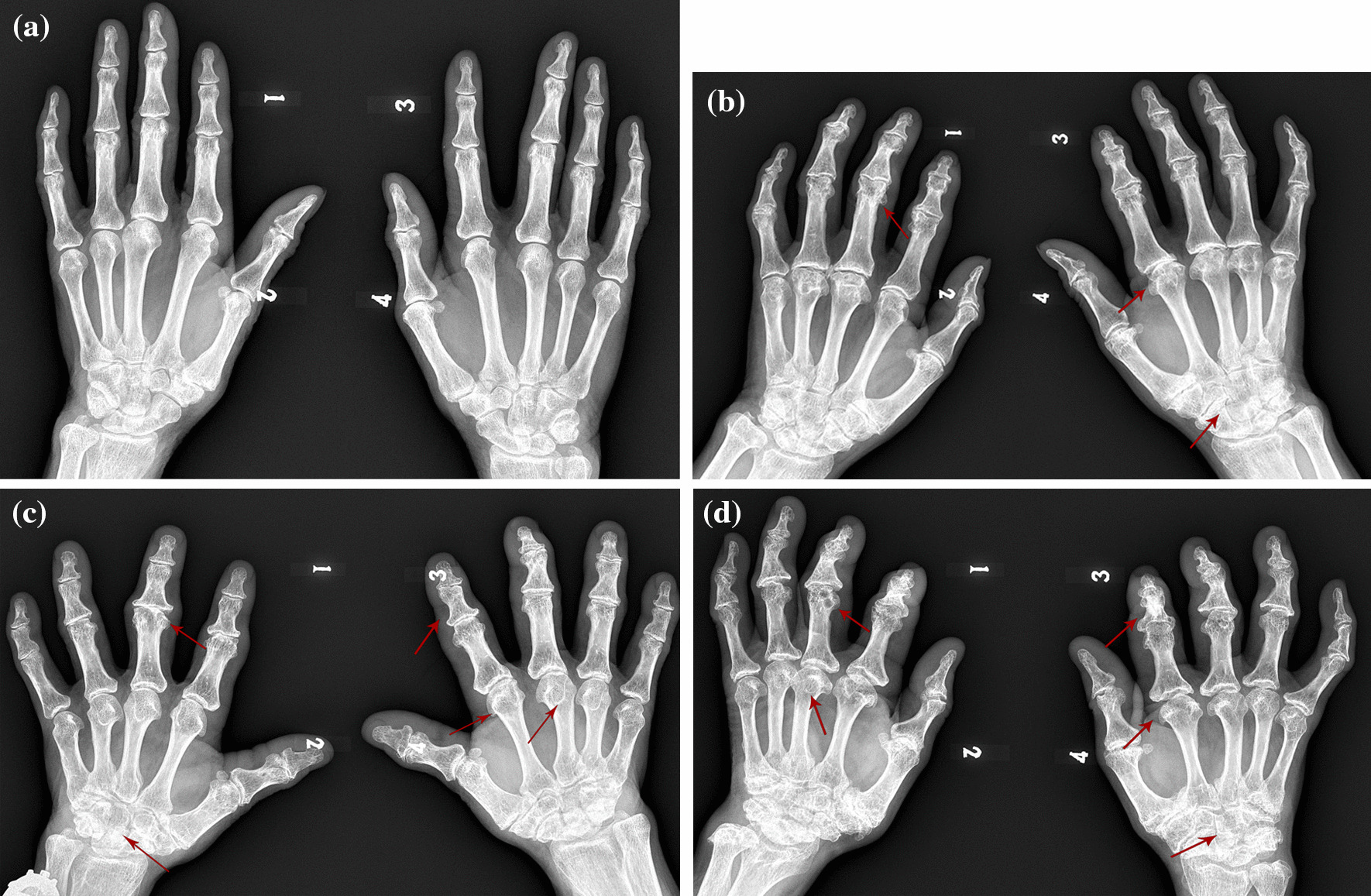
Posterior and anterior X-ray images of both hands in KBD patients and normal controls. **a** Normal adult hand X-ray image: smooth joint surfaces of palm, finger, wrist, and the joint space is symmetrical and clear. **b** Hand X-ray images of patients with stage Ӏ: formation of ridge in the middle phalanx, hypertrophy of the tuberosity; pseudocyst of the metacarpal head; crowding and deformation of the carpal bone. **c** Hand X-ray images of patients with grade II: shortening of the metacarpal phalanx, widening of the base of the phalanx in a triangular shape, and hyperplasia of the edge; swelling of the metacarpal head, pseudocyst, and falciform shadow; crowding and deformation of the carpal bone. **d** Hand X-ray images of patients with grade III: metacarpophalangeal shortening, widening of the base of the phalanx, bell-shaped changes at the proximal end, hyperplasia of the edge, and articular surface coloboma; metacarpal head swelling, falciform shadow; carpal bone crowding and deformation

Among the 8 surveyed counties (cities, districts), a total of 1309 valid samples were collected. A total of 710 patients with KBD were detected, including 332 males with an average age of (58.71 ± 7.64) years old and 378 females with an average age of (57.05 ± 6.96) years old. A total of 245 patients with grade I were detected, with a detection rate of 18.7%; 175 patients with grade II, with a detection rate of 13.4%; 25 patients with grade III, with a detection rate of 1.9%; 265 with atypical patients, the detection rate was 20.2%. In severe endemic area of KBD, the detection rates of grade I, grade II, grade III, and atypical KBD were 34.0%, 19.5%, 2.5%, and 26.7%, respectively. In moderate endemic area of KBD, the detection rates of grade I, grade II, grade III, and atypical KBD were 9.7%, 18.0%, 2.4%, and 15.7%, respectively. In mild endemic area of KBD, the detection rates of grade I, grade II, grade III, and atypical KBD were 8.1%, 1.2%, 0%, and 12.1%, respectively (Table 2). There was no significant difference in the detection rate of grade I KBD and atypical KBD (*χ*^2^ = 0.974,* P* > 0.05). Different grades of KBD patients showed different degrees of hand X-ray appearances (Fig. 1).

**Table 2:** Epidemiological survey results of Kashin–Beck disease in 8 counties (cities, districts) (number of people)

Areas	Counties	Number	Diagnostic result [number of people, detection rate (%)]				
			Normal	Stage I	Grade II	Grade III	Atypical
Severe endemic area	Yanshou	124	78 (62.9)	60 (4.8)	14 (11.3)	0 (0.0)	26 (21.0)
	Fuyu	419	64 (15.3)	127 (30.3)	106 (25.3)	15 (3.6)	107 (25.5)
	Shangzhi	225	45 (20.0)	74 (32.9)	30 (13.3)	4 (1.8)	72 (32.0)
Moderate endemic area	Tieli	138	93 (67.4)	11 (8.0)	9 (6.5)	0 (0.0)	25 (18.1)
	Bei'an	56	36 (64.3)	5 (8.9)	9 (16.1)	1 (1.8)	5 (8.9)
	Ning'an	54	27 (50.0)	8 (14.8)	5 (9.3)	5 (9.3)	9 (16.7)
Mild endemic area	Acheng	174	137 (78.7)	14 (8.0)	2 (1.1)	0 (0.0)	21 (12.1)
Non-KBD area	Qian'an	119	119 (100.0)	0 (0.0)	0 (0.0)	0 (0.0)	0 (0.0)
	Total	1 309	599 (45.8)	245 (18.7)	175 (13.4)	25 (1.9)	265 (20.2)

### Detection of ankle joint changes in different KBD areas

Among the surveyed 1309 people, 664 were found to have ankle joint changes, the detection rate was 50.7%. The detection rates of ankle joints in the severe, moderate, mild endemic area of KBD and non-KBD area were 70.0%, 35.1%, 17.2%, and 9.2%, respectively. The detection rate in severe endemic area of KBD was the highest, while the detection rate in non-KBD area was the lowest. The difference in the detection rate of ankle joint changes between the severe endemic area and the moderate endemic area was statistically significant (*χ*^2^ = 95.227, *P* < 0.01). The difference in the detection rate of ankle joint changes between the moderate endemic area and the mild endemic area was statistically significant (*χ*^2^ = 16.240, *P* < 0.01)(Table 3).

**Table 3:** Detection of ankle joint changes in different wards

Areas	Number of people checked	Number of ankle changes	Ankle change rate (%)
Severe endemic area	768	536	70.0
Moderate endemic area	248	87	35.1
Mild endemic area	174	30	17.2
Non-KBD area	119	11	9.2
Total	1309	664	50.7

### Rate of ankle joint changes in different grade of KBD

The patients with KBD were screened according to whether there were changes in the ankle joint. It was found that among all the investigated patients, 92.0% of the patients had ankle joint changes. 8.0% of the patients had no significant changes in the ankle joint. 80.0% of grade I patients had ankle joint changes. 95.4% of grade II patients had ankle joint changes. Both grade III and atypical patients had ankle joint changes (Table 4).

**Table 4:** Rate of ankle joint change of patients with KBD in each grade

Grade	Number of cases	Ankle changes [People, the detection rate (%)]	
		Changed	No change
I	245	196 (80.0)	49 (20.0)
II	175	167 (95.4)	8 (4.6)
III	25	25 (100.0)	0 (0.0)
Atypical	265	265 (100.0)	0 (0.0)
Total	710	653 (92.0)	57 (8.0)

### Degree of ankle joint change in X-ray

**Fig. 2:**
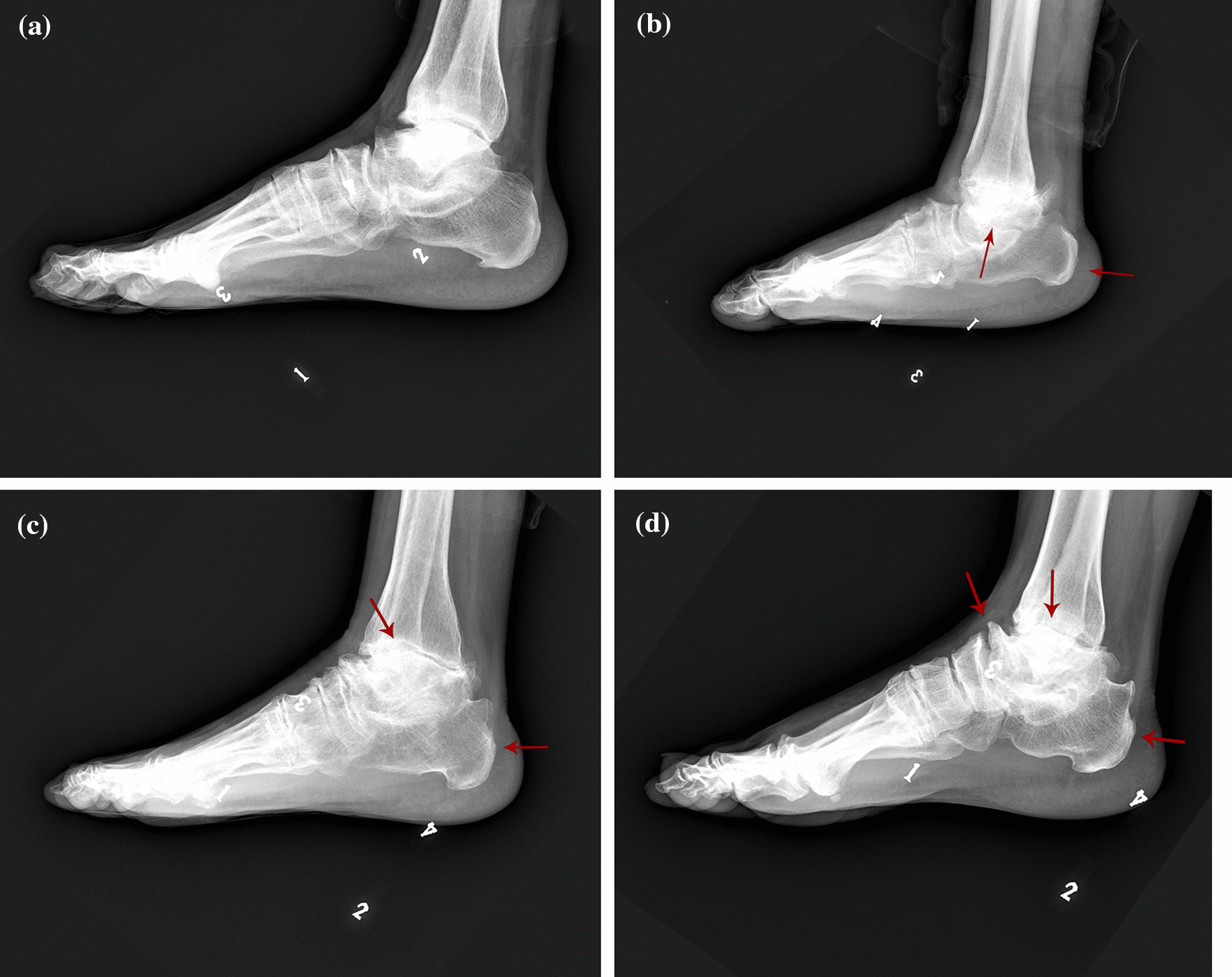
X-ray images of lateral ankle joint in patients with KBD and normal controls. **a**Normal adult ankle joint X-ray image: normal calcaneus length, no hyperplasia of the talar head and body, and smooth articular surface of the talus. **b** X-ray image of degree I ankle joint changes: the calcaneus is slightly shorter, the talar head and body are hyperplasia, and the articular surface of the talus is smooth. **c** X-ray image of degree II ankle joint changes: the calcaneus is slightly shorter, the posterior and superior border of the calcaneus is hyperplasia, the head and body of the talus are hyperplasia, and the articular surface of the talus is rough and uneven. **d** X-ray image of degree III ankle joint changes: short calcaneus, hyperplasia of talus head and talus body, upturned talus head and navicular bone to form bone spurs and peripheral cuneiform spurs, talus articular surface collapsed and flattened

Ankle joint X-rays of 710 patients with KBD were read and graded according to pre-set grading standards (Fig. 2). After statistical test, the severity of ankle joint changes in patients with grade II was higher than grade I (*χ*^2^ = 56.208,*P* < 0.01). The severity of ankle joint changes in patients with grade III was higher grade II (*χ*^2^ = 11.879,*P* < 0.01). There was no significant difference between patients with grade I and atypical patients (*χ*^2^ = 2.613,*P* < 0.05). The degree of ankle joint changes was correlated with the stage of KBD (*P* < 0.001). Correlation coefficient *r*_*s*_ = 0.376(Table 5).

**Table 5:** The degree of ankle joint changes and the classification of KBD

Degree	X-ray diagnosis [person, detection rate (%)]			
	Grade I	Grade II	Grade III	Atypical
Normal	49 (20.0)	8 (4.6)	0 (0.0)	0 (0.0)
Degree I	97 (39.6)	38 (21.7)	0 (0.0)	113 (42.6)
Degree II	63 (25.7)	59 (33.7)	7 (28.0)	90 (34.0)
Degree III	36 (14.7)	70 (40.0)	18 (72.0)	62 (23.4)
Total	245 (100.0)	175 (100.0)	25 (100.0)	265 (100.0)

### Age distribution of patients in different KBD areas

After statistical test, the patients with atypical KBD in the mild endemic area were younger than typical KBD (*P* < 0.05). There was no significant difference in age between patients with typical KBD and atypical KBD in the moderate endemic area (*P* > 0.05). The patients with atypical KBD in the severe endemic area were younger than typical KBD (*P* < 0.05) ( Table 6).

**Table 6:** Age distribution of typical and atypical Kashin–Beck disease patients in different wards [*M*(*P*25 –*P*75)]

Areas	Typical	Atypical	*Z*	*P*
Mild endemic area	61.00 (56.50–64.75)	57.00 (53.50–60.50)	− 1.982	0.048
Moderate endemic area	55.50 (50.25–61.00)	54.50 (49.00–62.25)	− 0.707	0.479
Severe endemic area	60.00 (54.0 –64.00)	55.50 (51.00–62.00)	− 4.718	0.000

## Discussion

### Rate of ankle joint changes in patients with KBD

The morphological changes of osteochondral in KBD are complicated. Because the patient's age, involved location, nature of the disease, and severity are different, there will be a variety of X-ray signs in different bone and joint parts. In KBD adults, a severe decrease in activity is primarily caused by a decrease in range of joint motion, rather than by age, gender, muscle strength, and the presence of pain [1]. KBD is more common in the distal appendicular skeleton; that is, fingers, wrists, and ankles are more likely to be affected than hips and shoulders [17]. A study found that, for 118 KBD patients, eighty-one patients (68.6%) had abnormal ankle radiographs; 72 (88.9%) patients had talus changes, 69 (85.2%) patients had calcaneus changes, 28 (34.6%) patients had navicular bone changes, and 48 (59.2%) patients had distal tibia changes [18]. Another study found that, among 105 KBD cases in Tibet, 89.5% of KBD cases involved the feet and ankles [19]. Based on a 2001 study conducted on 2560 subjects in China’s Shaanxi Province, 69.3% of participants in this study showed limited ankle motion [8]. This study analyzed 710 cases of adult KBD patients with ankle changes, and found that not all patients have ankle changes. In this study, 80.0% of grade I patients, 95.4% of grade II patients, and all grade III patients had ankle joint changes. Previous studies have shown that the first site of KBD is the metaphysis of the hand and finger (toe), followed by the end of the bone, epiphysis, wrist and tarsal, and finally spread to the elbow, ankle, knee, shoulder, hip, spine, etc. It begins with smaller joints in the early grades of disease, over time, larger joints become involved [13]. Therefore, we speculated the reason for the patient with no change in the ankle joint is that the patient was in the early grade of onset. At this time, the hand has damaged with the risk factor, but the ankle joint has not yet undergone characteristic changes.

### Changes of ankle joint in different KBD areas

The trend of ankle joint changes detection rate was the same as hand. The detection rate was the lowest in non-KBD areas and the highest in severe endemic areas. It increased with the severity of the disease in the ward. It shows that the ankle joint changes can also be used as a specific indicator for the diagnosis of KBD. This study involved non-KBD area, mild endemic area, moderate endemic area and severe endemic area, we can speculate the situation of KBD patients’ ankle joints changes in various wards across the country.

### Classification of degree in ankle joint change

The previous X-ray diagnosis of KBD mainly focused on the hand joints. But there was no detailed study on the changes of ankle joints. The imaging findings and related data of the ankle are different between the patients with KBD and normal people, which can be used as an auxiliary diagnostic index of KBD. Previous studies have suggested that KBD is characterized by a short calcaneus and a collapsed talus. According to the classification of the X-ray changes of the talus and calcaneus of the foot in the diagnosis standard of KBD (WS/T 207–2010), combined with the symptoms reflected in this study, the degree of changes in the ankle joint was divided into three grades, and applied to the research. In this study, the degree of ankle joint change was graded, and it was found that there was a correlation between the degree of ankle joint change and the grade of KBD. The higher the grade of KBD, the more severe the ankle joint change. In the current diagnostic criteria of KBD, there is no detailed classification of ankle changes. In this study, a new grading method of ankle joint changes was proposed by reviewing the X-ray films of a large number of patients with KBD, combining with many years' experience in diagnosis of KBD. Grading the degree of ankle joint changes in KBD is helpful to the differential diagnosis and further study of KBD.

### Atypical KBD

The KBD cases existing in China are old cases with secondary clinical signs of osteoarthritis, including typical and atypical cases. The diagnosis standard of KBD (WS/T 207–2010) defined typical cases as having multiple symmetrical thickened knuckles, deformities and even short stature. The KBD pathogenic factor destroys epiphyseal plate in children before the closure of tubular diaphysis epiphysis [20]. The typical clinical cases of KBD are all occurred in childhood. It is because adults have completed their epiphyseal development, and no longer appear brachydactyly or dwarf physique even if they are hit by pathogenic factors, whether locals or immigrants. Unlike typical KBD patients, atypical KBD patients have no characteristic changes in the hand and need to be diagnosed by characteristic changes in the calcaneus and talus of the ankle.

This study found that 100% of typical KBD patients with stage III had shorten calcaneal and talus collapse. Therefore, the collapse of the ankle talus and shorten calcaneus can be defined as characteristic changes of KBD. The results of this study showed that there was no significant difference in the detection rate and the degree of ankle joint change between the atypical patients and the patients with grade I. It can be considered that the degree of joint damage in atypical patients and the patients with grade I is similar, but the damage sites are different.

### Causes of becoming atypical Kashin–Beck disease

Adults with KBD always occur in childhood. So it is important to link bone and joint damage to the process of bone development. Zhang et al. [21] proposed that age of the epiphyseal closure in metacarpophalangeal bones of males was from 14 to 16 years old, and in females was 13 to 15 years old, while age of the secondary ossification center closure in calcaneal bones is 16 to 18 years old in males and 14 to 16 years old in females. The epiphyseal closure age was 15.0–17.0 years in women and 16.0–18.0 years in men. Therefore, we considered that the epiphyseal closing time of metacarpal and phalangeal epiphysis is earlier than ankle joint and calcaneal epiphysis. When the children in endemic areas hit by the causative factors of KBD, they were in the period of that the epiphysis of metacarpophalangeal and phalangeal were closed, but the epiphysis of calcaneal were not closed.

Another important factor in the development of atypical KBD is the facilitation of gravity. Studies have shown that weight load has a major impact on the musculoskeletal system associated with degeneration and inflammation, especially osteoarthritis [22]. The joint damage of KBD is similar to osteoarthritis. With small doses of risk factor, KBD causes no significant characteristic changes in the metacarpophalangeal joint, whereas the ankle joint is the main load-bearing joint of the human body. When struck by the same dose of pathogenic factors, the overlapping effect of pathogenic factors and gravity will aggravate the injury of articular cartilage, resulting in the characteristic changes such as talar hyperplasia, talar joint roughness, concavity and collapse, atypical KBD.

We hypothesized that atypical KBD occurred at the end of the epidemic period. Comparing the age distribution with different degrees, we found that the patients with atypical KBD in the mild and severe endemic areas were younger than typical KBD. Since the typical patient was born at the peak of the KBD epidemic, the age of the atypical patient was younger than that of the typical patient. It can be inferred that the intensity of the pathogenic factors in the area had weakened and was at the end of the epidemic period, resulting in less joint damage in the atypical patient, and only the ankle joint was changed. The intensity of pathogenic factors in mild disease area is weak. And the epidemic peak is not obvious. Therefore, there is no difference in age distribution between atypical and degree I patients. But the intensity of pathogenic factors in the serious disease area is high, and the difference in age is significant.

## Conclusions

In conclusion, 80.0% of grade I patients, 95.4% of grade II patients, and all grade III patients had ankle joint changes. The higher the grade of KBD, the more severe the ankle joint change. We speculate that the formation of atypical Kashin–Beck disease is related to the closure time of the epiphysis and the effect of gravity, and the atypical Kashin–Beck disease occurs at the end of the epidemic grade of Kashin–Beck disease.

## Data Availability

The datasets used and analyzed during the current study are available from the corresponding author upon reasonable request.
