# Correction: A study on atypical Kashin–Beck disease: an endemic ankle arthritis

**DOI:** 10.1186/s13018-023-03860-z

**Published:** 2023-05-23

**Authors:** Fang Qi, Si-Lu Cui, Bing Zhang, Hao-Nan Li, Jun Yu

**Affiliations:** 1 Institute for Kashin‑Beck Disease Control and Prevention, Chinese Center for Disease Control and Prevention, Harbin Medical University, Harbin, 150081 Heilongjiang China; 2 National Health Commission and Education Bureau of Heilongjiang Province, Key Laboratory of Etiology and Epidemiology, Harbin Medical University(23618504), Heilongjiang Provincial Key Laboratory of Trace Elements and Human Health, Harbin Medical University, Harbin, 150081 China

**Correction: Journal of Orthopaedic Surgery and Research (2023) 18:328** **https://doi.org/10.1186/s13018-023-03633-8**

Following publication of the original article [1], the authors identified an error in the funding note.

The correct funding note is given below.

This work was supported by: (1) National Natural Science Foundation of China (No. 81972983); (2) National Key Research and Development Program of China (2022YFC2503101).

The original article [1] has been corrected.
